# ABCA6 Regulates Chondrogenesis and Inhibits Joint Degeneration via Orchestrated Cholesterol Efflux and Cellular Senescence

**DOI:** 10.1002/advs.202410414

**Published:** 2025-01-17

**Authors:** Yi Wang, Qiang Wu, Yongqing You, Wenbo Jiang, Peiliang Fu, Kerong Dai, Ye Sun

**Affiliations:** ^1^ Department of Orthopaedics The First Affiliated Hospital of Nanjing Medical University Jiangsu 210029 China; ^2^ Shanghai Key Laboratory of Orthopaedic Implants Department of Orthopaedic Surgery Shanghai Ninth People's Hospital Shanghai Jiao Tong University School of Medicine Shanghai 200011 China; ^3^ Renal Division Affiliated Hospital of Nanjing University of Chinese Medicine Nanjing 210008 China; ^4^ Clinical and Translational Research Center for 3D Printing Technology Shanghai Ninth People's Hospital Shanghai Jiao Tong University School of Medicine Shanghai 200011 China; ^5^ Department of Orthopaedics Shanghai Changzheng hospital Naval Medical University Shanghai 200003 China

**Keywords:** ABCA6, cellular senescence, cholesterol efflux, chondrogenesis, osteoarthritis, patellar dysplasia

## Abstract

Patellar dysplasia (PD) can cause patellar dislocation and subsequent osteoarthritis (OA) development. Herein, a novel ABCA6 mutation contributing to a four‐generation family with familiar patellar dysplasia (FPD) is identified. In this study, whole exome sequencing (WES) and genetic linkage analysis across a four‐generation lineage presenting with six cases of FPD are conducted. A disease‐causing mutation in ABCA6 is identified for FPD. Further analyses reveal a consistent correlation between ABCA6 expression downregulation and PD occurrence, chondrocyte degeneration, and OA onset. Moreover, ABCA6‐KO mice demonstrate severe knee joint degeneration and accelerated OA progression. Besides, synovial mesenchymal stem cells (SMSCs) are extracted from WT, ABCA6^−/+^, and ABCA6^−/−^ mice to create chondrogenic organoids in vitro, confirming ABCA6 deficiency can lead to chondrocyte degeneration via modulating cell cycle and activating cellular senescence. Moreover, transcriptome and metabolomic sequencing analysis on ABCA6‐KO chondrocytes unveils that the ABCA6 deficiency inhibits cholesterol efflux, leading to intracellular cholesterol accumulation and subsequent cellular senescence and impaired chondrogenesis.A disease‐causing mutation of ABCA6 is identified for FPD. ABCA6 is correlated with PD occurrence and subsequent OA progression. ABCA6 can serve as a potential target in chondrogenesis and OA treatment by orchestrated intracellular cholesterol efflux and delayed cellular senescence.

## Graphical abstract

Patellar dysplasia may cause patellar dislocation and osteoarthritis (OA). A novel ABCA6 mutation in a four ‐ generation family is identified. ABCA6 is related to patellar dysplasia and OA progression. Its downregulation correlates with OA onset. ABCA6 regulates chondrogenesis and inhibits joint degeneration via orchestrated cholesterol efflux and cellular senescence, therefore becoming a potential target for chondrogenesis and OA treatment.

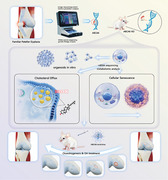

## 1 Introduction

Patellar dysplasia is one risk factor causing patellar dislocation and subsequent osteoarthritis development.^[^ ^1^, ^2^, ^3^, ^4^ ^]^ It has been reported as a feature in many genetic disorders such as small patella syndrome and nail patella syndrome, and studies on these disorders have implicated that several genes were involved in patellar dysplasia.^[^ ^5^, ^6^ ^]^ Bongers et al.^[^ ^7^ ^]^ found that TBX4 was associated with small patella syndrome, the disease that is characterized mainly by patellar reduction or absence in six families through genetic association analysis and sequencing. Study by Dreyer et al.^[^ ^8^ ^]^ suggested that alteration of LMX1B function in mice and human resulted in similar phenotypes like nail‐patella syndrome characterized by absent patellae and hypoplastic nails. Marilyn et al.^[^ ^9^ ^]^ identified WIF1 as a potential novel cause of a Nail‐Patella‐like disorder in a four‐generation family. Moreover, it was reported that RAPADILINO syndrome, which especially affects bone development commonly for the forearms and the patellae, could be caused by the mutation of RECQL4.^[^ ^10^, ^11^ ^]^ However, genetic heterogeneity is found among these diseases. For instance, Bongers et al.^[^ ^12^ ^]^ did not identify mutations of LMX1B in 4 out of 32 families diagnosed with nail‐patella syndrome. As with or RAPADILINO syndrome, mutations of RECQL4 were found only in 46% of the patients receiving gene mutation screening.^[^ ^11^ ^]^ From these facts, it evidently implies that multiple genes were related to patellar dysplasia.

ABCA, the A‐family of the ATP binding cassette transporter, is responsible for mediating transmembrane transport of various molecules. Besides, its family members like ABCA4, ABCA8, and ABCA9, have been reported in skeletal development. In animal study, ABCA4 knockout mice showed obvious defects in skeletal development with reduced trabecular number, decreased bone content, and decreased bone mineral density.^[^ ^13^ ^]^ In epidemiological study, ABCA8 expression levels were reported to be significantly correlated with the bone density in postmenopausal women.^[^ ^14^ ^]^ ABCA9 is confirmed to be the pathogenic gene leading to a rare genetic disease named Cantu syndrome characterized by osteo‐chondrodysplasia (thickening of the calvaria, broad ribs, scoliosis, and flaring of the metaphyses).^[^ ^15^, ^16^ ^]^ As for ABCA6, although cholesterol reactivity was suggested for ABCA6 variants,^[^ ^17^ ^]^ its impacts on skeletal development disorders were lacking. Consequently, relevant mechanism of transcriptional regulation and biological function on bone development are worth further researching.

Herein, we present the genetic findings about a four‐generation Chinese family with familiar patellar dysplasia (FPD, No. patient = 6) by utilizing integrated approaches including whole exome sequencing (WES), linkage analysis, Sanger sequencing, and protein function analysis. We have identified a novel ABCA6 mutation contributing to patellar dysplasia. Further experiments were conducted to delve into the role of ABCA6 in chondrogenesis and OA progression.

## 2 Result

### 2.1 WES and Protein Function Analysis Indicate the Mutation of ABCA6 as a Causative Gene of Familiar Patellar Dysplasia

**Figure 1:**
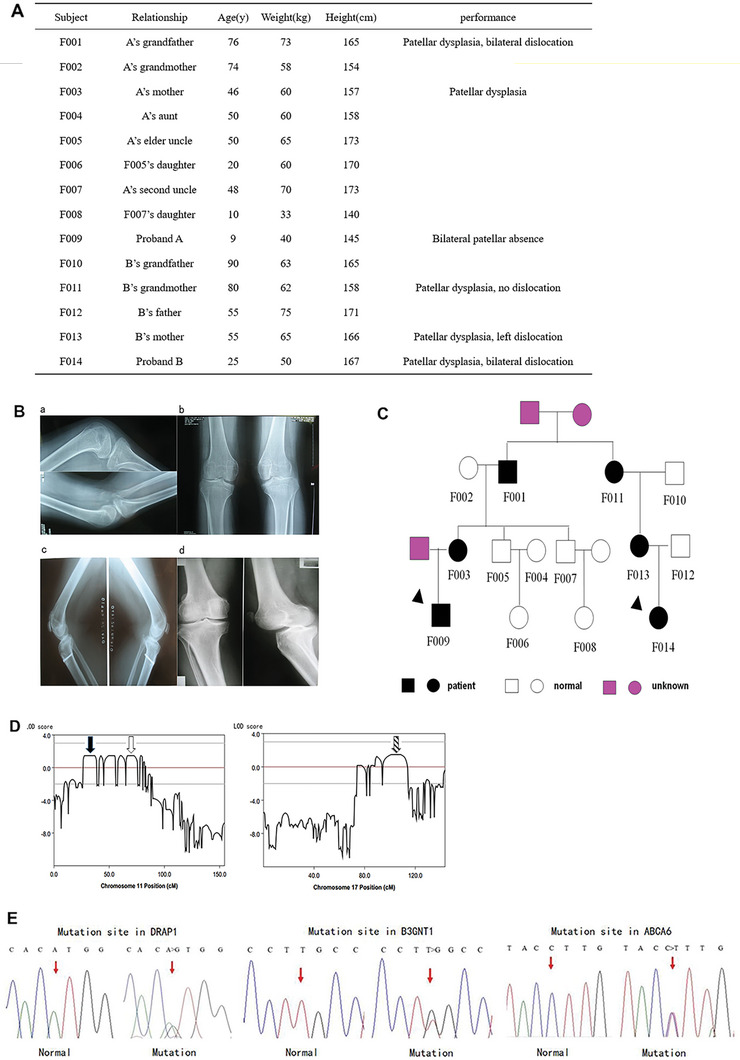
WES and protein function analysis indicate ABCA6 as a causative gene of FPD. A) General information of all family members in the FPD family. B) Radiological X‐ray Images of patellar dysplasia of proband A, his mother and his grandfather. a,b) Lateral and anteroposterior radiographs show bilateral patellar absence of proband A. c) Lateral radiographs show bilateral small patella combined with patellar dislocation of the proband's mother. Patellar diaplasis was performed for this woman. d) Lateral and anteroposterior radiographs show left small patella combined with patellar dislocation of the proband's grandfather. C) Pedigree of the four‐generation family with a dominantly inherited patellar dysplasia. The probands are indicated by the arrows. Family members F009, F003, and F005 underwent WES. D) Linkage association study showed several regions with LOD > 1 in chr11 and chr17. Combined results of WES and linkage studies identified DRAP1 (solid arrow) and B3GNT1 (hollow arrow) in chr11 (a) and ABCA6 (striped arrow) in chr17 (b) as candidate genes. E) Sanger sequencing verified three mutations in DRAP1 (a), B3GNT1 (b), and ABCA6 (c) respectively in all study family members and 200 independent Chinese normal controls. All three mutations were heterozygous missense mutations in all affected family members.

We have stumbled upon a FPD family of a four‐generational lineage, wherein six instances of patellar dysplasia have been identified (**Figure** **1**A). Diagnosis of the proband (F009) and his relatives were based on radiographs and medical history (Figure 1B). The family pedigree is shown in Figure 1C.

WES was performed for two patients and one normal control in the FPD family. One hundred and eleven new mutation sites were identified (details are shown in Table S1, Supporting Information), including six mutation homozygote and 105 mutation heterozygote. Of them, there were 85 missense mutation, 12 non‐frameshift deletion mutation, six frameshift deletion mutation, four non‐frameshift insertion mutation, two non‐frameshift transition mutation, one early termination mutation, and one frameshift insertion mutation. No mutations in gene TBX4, LMX1B, and RECQL4 were identified in the WES stage. Genetic linkage analysis was performed in all family members and the chromosomal regions with linkage of disequilibrium (LOD) >1 were identified in chromosome 11 and 17 (Figure 1D). In combination with the results of WES, six mutation sites were confirmed respectively in B3GNT1, DRAP1, ABCA10, ABCA10, ABCA6 and SYNJ1 (Table S2, Supporting Information). In situ Sanger sequencing was performed for all family members and six mutations were identified. One mutation site located on SYNJ1 has been excluded through in situ verification. Two mutation sites in ABCA10 were excluded as polymorphism sites. The remaining three mutations respectively resulting in the amino acid changes of B3GNT1 K334Q, DRAP1 M102V, and ABCA6 D797N were not detected in the 200 independent Chinese normal controls.

The three heterozygous missense mutation sites were detected in all patients with patellar dysplasia, but were not detected in any normal samples in the study family or the 200 independent Chinese normal controls, suggesting that DRAP1 M102V, B3GNT1 K334Q, and ABCA6 D797N could be the pathogenic genes responsible for patellar dysplasia in this family (Figure 1E). DRAP1 M102V (SNP rs543738162) and B3GNT1 K334Q (SNP rs557718654) were identified as polymorphism sites but previously unreported in Chinese. The frequency of rs543738612 and rs557718654 were both 0.02% in global and 0.1% in East Asians. However, this study was the first to show these two mutations in Chinese population. The mutation site in DRAP1 caused changes in amino acid sequence of M102V, and cross‐comparison with variant organisms showed the mutation site was located in the conservative area of the protein product of DRAP1. The following prediction of protein function showed the induced effect was harmless. The same analysis procedure was performed for B3GNT1 and it showed the caused change of amino acid K334Q had no negative effect on the ultimate protein function. The mutation site ABCA6 D797N was demonstrated to be a splice site of ABCA6 protein and potentially disease causing (Table S3, Supporting Information). This suggested that the mutation of ABCA6 was the most likely causative factor of patellar dysplasia in the study family.

### 2.2 ABCA6 Expression Was Correlated with Chondrocyte Degeneration and Subsequent Osteoarthritis in Patellar Dislocation Patients

**Figure 2:**
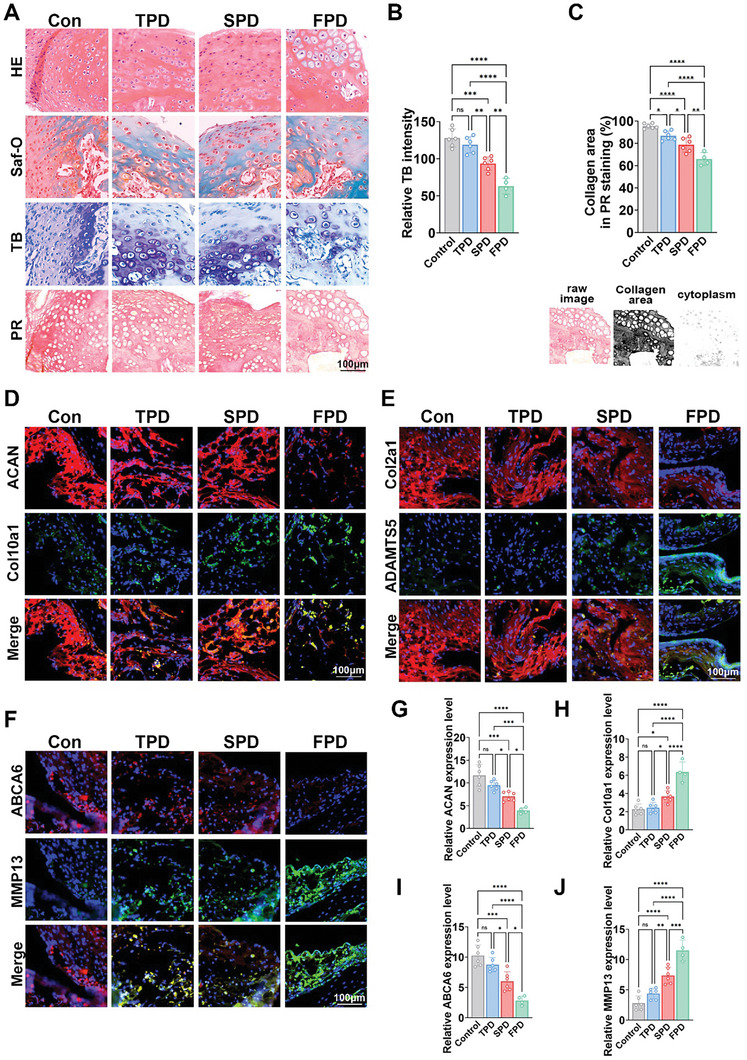
ABCA6 expression was correlated with chondrocyte degeneration and subsequent osteoarthritis in patellar dislocation patients. A) Histological analysis was conducted of the cartilage tissue sections with H&E (first row), saf‐O (second row), TB (third row), and PR (forth row) staining. B) Relative TB staining intensity in different group. **p < 0.01, ***p < 0.001, ****p < 0.0001, NS not significant, *n* = 6 in control, TPD, SPD and 4 in FPD. C) Quantification of collagen area in PR staining with different treatment groups. *p < 0.05, **p < 0.01, ***p < 0.001, ****p < 0.0001, *n* = 6 in control, TPD, SPD and 4 in FPD. The value is derived from the ratio of the collagen area to the area of the raw image. D–F) Immunofluorescent assay of ACAN (D: red), Col10a1(D: green), Col2a1(E: red), ADAMTS5(E: green), ABCA6(F: red), and MMP13(F: green) expression and nucleus in different groups. G–J) Relative ACAN(G), Col10a1(H), ABCA6(I), and MMP13(J) genes expression levels in different groups of immunofluorescent assay. *p < 0.05, **p < 0.01, ***p < 0.001, ****p < 0.0001, NS not significant, *n* = 6 in control, TPD, SPD and 4 in FPD.

In order to investigate the correlation between ABCA6 and patellar dislocation and consequent osteoarthritis (OA), we conducted histomorphological analysis on the cartilage samples of knee joint from patients with traumatic patellar dislocations (TPD), sporadic patellar dislocations (SPD), and familial patellar dislocations (FPD). Hematoxylin‐eosin (HE) staining, safranin‐O/Fast green (Saf‐O) staining, toluidine blue (TB) staining, and picrosirius red (PR) staining were employed (**Figure** **2**A). Upon all three types of patella dislocation in our studies, similar osteoarthritic changes were observed, including hypertrophic changes in chondrocytes, decreased cartilage matrix precipitation, reduced collagen fibers, and an increase in cartilage hypertrophy and cartilage decomposition (Figure 2B,C). Moreover, FPD cartilage samples demonstrated significantly lower chondrogenic ECM deposition and much less collagen area compared to other groups (Figure 2B,C). Immunofluorescence staining further showed a decline in ACAN and Col2a1 expressions, but elevated levels of Col10a1, ADAMTS5, and MMP13 in patellar dislocation specimens (Figure 2D–H,J). Concurrently, ABCA6 expression was also significantly reduced in all patellar dislocation samples, especially in the FPD cartilage samples (Figure 2F,I). Based on these findings, we hypothesize that the diminution or absence of ABCA6 gene expression could cause cartilage degeneration by impacting the chondrocyte anabolism and catabolism, leading to patella underdevelopment and the subsequent osteoarthritic changes.

### 2.3 ABCA6‐KO Mice Demonstrated Severe Knee Joint Degeneration and Significant Accelerated OA Progression

**Figure 3:**
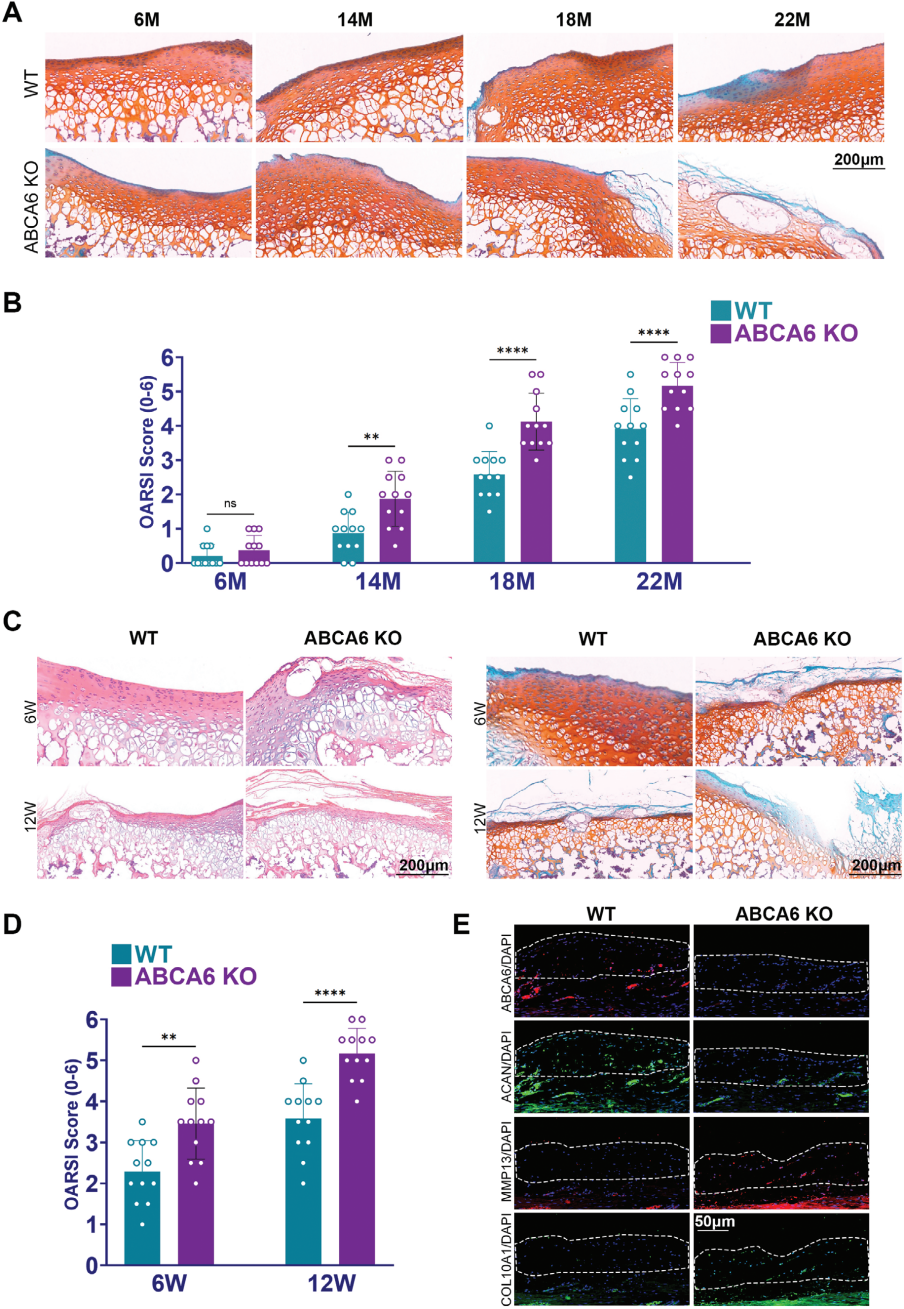
ABCA6‐KO mice demonstrated severe knee joint degeneration and significant accelerated OA progression. A) Saf‐O staining of knee joint cartilage tissue in wild type mice and ABCA6 knock out mice at the 6th, 14th, 18^th^, and 22th month. B) OARSI score of ABCA6 knock out and wild type mice over 22 months. *n* = 12 for each. **p < 0.01, ****p < 0.0001, NS not significant. C) H&E and Saf‐O stainings of knee joint cartilage tissue in surgically induced OA in ABCA6 knock out and wild type mice at the 6th and 12th week. D) OARSI score in surgically induced OA of ABCA6 knock out and wild type mice at the 6th and 12th week. *n* = 12 for each. **p < 0.01, ****p < 0.0001. E) Immunofluorescence staining of ABCA6, ACAN, MMP13, and COL10A1 expression of knee joint cartilage tissue in surgically induced OA in ABCA6 knock out and wild type mice at the 12th week time point (red: ABCA6, MMP13; green: ACAN, COL10A1; blue, DAPI).

In order to substantiate our hypothesis, we obtained ABCA6 knockout (ABCA6‐KO) mice and observed them over a period of 22 months. Successful knockout of ABCA6 in mice was identified with WB staining for ABCA6 expression, and the Saf‐O & fast green staining of the whole body skeleton was performed for neonatal ABCA6‐KO mice, demonstrating similar body length and normal bone and joint formation compared to WT mice (Figure S1, Supporting Information). During the 22‐month timeframe, in comparison to wild‐type (WT) mice, the ABCA6‐KO mice exhibited significantly more pronounced degeneration of knee joint cartilage, as demonstrated with Saf‐O staining, signaling the spontaneous development of OA in ABCA6 deficiency (**Figure** **3**A). Furthermore, the OA Severity Index (OASRI) scores for the ABCA6‐KO mice were notably higher than those for the WT group over the 22‐month period (Figure 3B). To corroborate the pivotal role of ABCA6 deficiency in OA development, we surgically induced OA in both WT and ABC6‐KO mice and monitored them for 12 weeks. Subsequently histological examination of the knee joint cartilage was carried out using H&E and Saf‐O staining (Figure 3C). Compared to WT mice, the ABCA6‐KO mice showed more severe degenerative alterations, greater cartilage tissue damage, fewer chondrocytes, and lower cartilage matrix deposition. This observation was consistent with the differences in OARSI scores between WT and ABCA6‐KO mice, demonstrated as a significant increase of OARSI score in the ABCA6‐KO group (Figure 3D). Immunofluorescence staining further supported these findings, showing a decrease in ACAN expression as well as increased expression of COL10A1 and MMP13 in the ABCA6‐KO group compared to the WT group (Figures 3E and S2, Supporting Information). These findings indicated that ABCA6 deficiency could lead to spontaneous joint degeneration in mice and significant accelerated OA progression in the mice OA model.

### 2.4 ABCA6 Deficiency Is Detrimental to Chondrogenesis of the Organoids In Vitro with Premature Chondrocyte Senescence and Cartilage Degeneration

**Figure 4:**
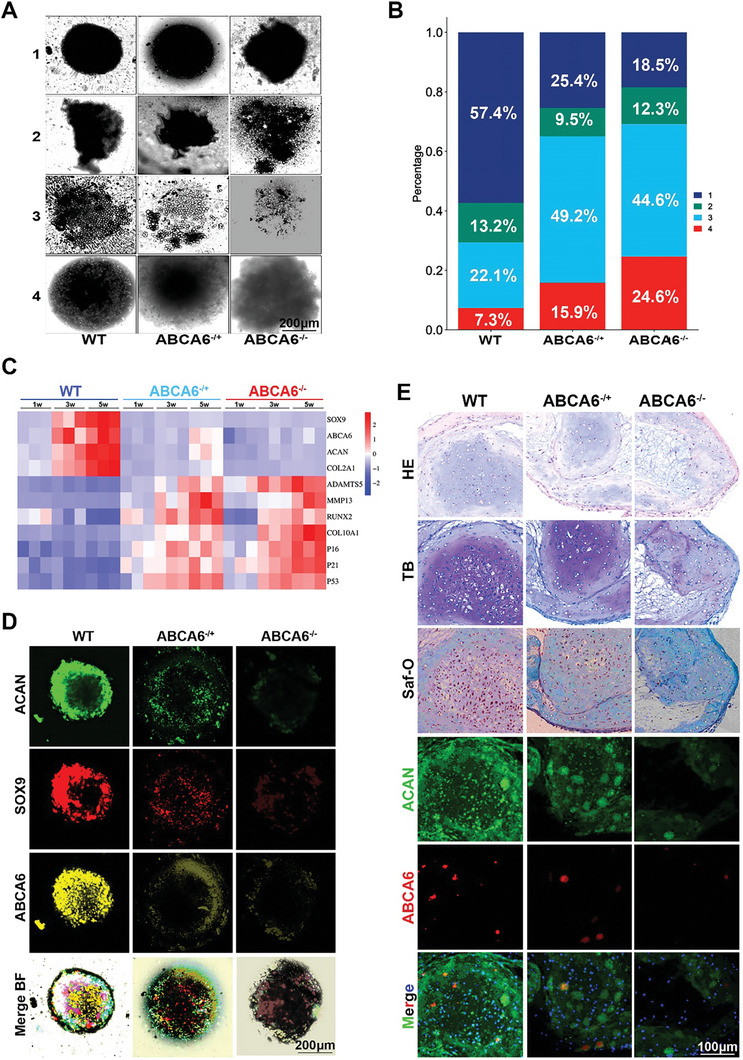
ABCA6 deficiency is detrimental to chondrogenesis of the organoids in vitro with premature chondrocyte senescence and cartilage degeneration. A) Microscopic images of the formed chondrogenic organoids from WT, ABCA6^−/+^, and ABCA6^−/−^ groups in the light field after 5 weeks of cultivation. B) Distribution of pellets in categories 1 to 4 of WT, ABCA6^−/+^, and ABCA6^−/−^ Groups. C) Quantification of gene expression in different groups with RT‐PCR for ABCA6, Chondrogenic, OA and senescence genes. D) Immunofluorescent assay in the category one of cartilage granules with ACAN (1st row), SOX9(2nd row), ABCA6(3rd row) expression in different groups and merge with Brightfield (4th row). E) Representative images of different groups cartilage granules in category three, H&E (1st row), TB (2nd row), Saf‐O (3rd row) staining and IF of ACAN (4th row), ABCA6 (5th row), merge (6th row).

To elucidate the potential pathogenic mechanisms associated with the observed deficiency in ABCA6 gene expression, we mated ABCA6 KO mice with both WT mice and homologous ABCA6 KO mice, and extract Synovial Mesenchymal Stem Cells (SMSCs) from the offspring produced. We divide them into WT, ABCA6^−/+^, and ABCA6^−/‐^ groups and cultured them in a 3D chondrogenic organoid system for a duration of five weeks. In a previous study, Meulenbelt et al.^[^ ^18^ ^]^ classified various types of chondral organoids based on distinct morphological characteristics of their granules, specifically shape (round or protrusive) and matrix opaqueness. This classification scheme delineated four categories of organoids, and its efficacy in predicting subsequent chondrogenic potential was empirically validated. Adopting this established framework, we also categorized our chondral organoids into four distinct categories (**Figure** **4**A). The prevalence of spherical, dense granules in the WT group accounted for 57.4% of the total, whereas a reduction was seen to drop at ≈25.4% in the ABCA6^−/+^ group and further declined to 18.5% in the ABCA6^−/−^ group. Moreover, the abundance of expanded granules with a more translucent matrix increased in the ABCA6^−/+^ (49.2%) and ABCA6^−/−^ (44.6%) groups, posing a stark contrast with the WT group (22.1%) (Figure 4B). When considering the first category as “normal” granules and the rest three as “abnormal,” we observed an increased incidence of abnormal granules in both ABCA6^−/+^ and ABCA6^−/−^ groups. This suggests a dominant effect of ABCA6 on the formation of dense and spherical granules, indicating that chondrogenic differentiation of SMSCs is inhibited if ABCA6 is absent. Furthermore, RT‐PCR analyses conducted at 1, 3, and 5 weeks revealed elevated expressions of chondrogenesis markers SOX9, ABCA6, ACAN, and COL2A1 in the WT group, with an upward trend over time. In contrast, OA associated degenerative markers ADAMTS5, MMP13, RUNX2, and COL10A1, along with cellular senescence markers P16, P21, and P53, were significantly upregulated in the ABCA6^−/+^ and ABCA6^−/−^ groups, more prominently in the ABCA6^−/−^ group (Figure 4C). In summary, these data indicate that the absence of ABCA6 impairs chondrogenic potential and promotes the onset and progression of chondrocyte degeneration.

To further delineate the disparities between the chondrogenic granules formed by the WT, ABCA6^−/+^, and ABCA6^−/−^ groups, and to categorize the granules as normal or abnormal, histological and immunohistochemical analyses were conducted on Category 1 and 3 (Figure 4D,E). Compared to normal chondrogenic granules (Category 1, Figures 4D), abnormal one (Category 3, Figure 4E) exhibited a significant reduction in the expression of the chondrogenic marker gene ACAN, alongside a decrease in ABCA6 expression. Additionally, a downward trend in the expression of chondrogenic marker genes SOX9 and ACAN was observed across normal and abnormal granules in ABCA6^−/+^ and ABCA6^−/−^ groups. Multiple histological staining of the organoids indicated a decline in extracellular matrix precipitation within the chondrogenic granules formed by ABCA6^−/+^ and ABCA6^−/−^ groups compared to the WT group. Collectively, our study underscores the detrimental impact of ABCA6 deficiency on chondrogenic potential and the deposition of cartilaginous extracellular matrix, leading to premature chondrocyte senescence and cartilage degeneration of the formed chondrogenic organoids in vitro.

### 2.5 ABCA6 Deficiency Was Detrimental to Chondrocyte Function via Modulating Cell Cycle and Activating Cellular Senescence

**Figure 5:**
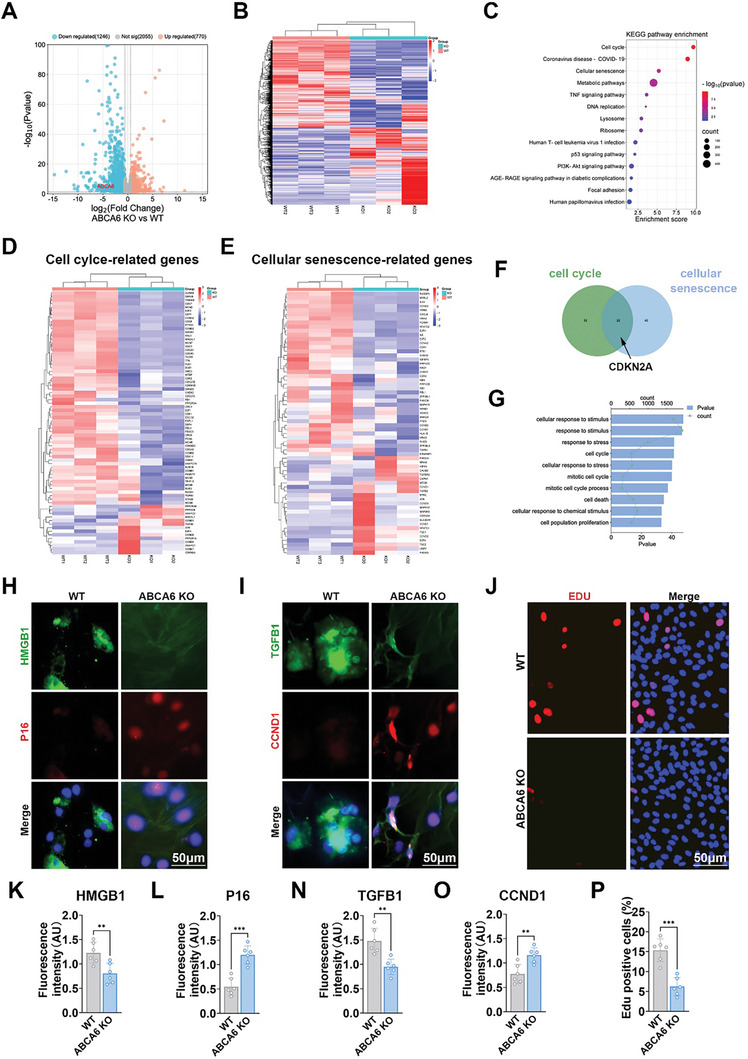
ABCA6 deficiency was detrimental to chondrocyte function via modulating cell cycle and activating cellular senescence. A) Volcano plot of mRNA expression profiles with microarray in WT group compared to ABCA6 KO group. B) Heatmap of clustering dysregulated mRNA expression profiles with microarray in WT group compared to ABCA6 KO group. C) TOP KEGG pathways enrichment differing in WT group compared to ABCA6 KO group. D,E) Heatmap of the expression profiles for cell cycle‐related genes (D) and cellular senescence‐related genes (E) in microarray expression profiles comparing the WT group to the ABCA6 KO group. F) Venn diagram of overlapping cell cycle‐related genes and cellular senescence‐related genes. G) GO Analysis of the CDKN2A Gene. H,I) HMGB1(H: green), P16(H: red), TGFB1(I: green), and CCND1(I: red) expressions assayed with fluorescent immunostaining. J) EdU Assay in WT group and ABCA6 KO group. K–P) Quantitative fluorescence intensity of HMGB1 (K), P16 (L), TGFB1 (N), CCND1 (O), and EDU (P). *N* = 6 in each group, **p < 0.01, ***p < 0.001.

In an endeavor to delve into the potential mechanisms yielding the degeneration of knee joints and the progression of OA in ABCA6 KO mice, we conducted mRNA sequencing analysis on chondrocytes derived from both WT and ABCA6‐KO mice. This analysis unveiled a distinct differential regulation of mRNAs in the ABCA6 KO group (**Figure** **5**A,B). Subsequent pathway analysis using Kyoto Encyclopedia of Genes and Genomes (KEGG) based on the entire differential gene expression profile (Figure 5C) revealed that in the ABCA6 KO group, there was a notable upregulation in pathways such as the cell cycle, cellular senescence, metabolic pathways, and the TNF signaling pathway. Most prominently, the cell cycle and cellular senescence pathways exhibited significant disparities. After that, we identified all genes related to these two pathways (Figure 5D,E), 22 genes were found to overlap (Figure 5F). Further gene ontology (GO) analysis of these genes, particularly CDKN2A (P16), highlighted functions predominantly associated with cell cycle regulation and cellular senescence (Figure 5G).

To provide a comprehensive assessment, we employed immunofluorescence staining to further evaluate both WT and ABCA6‐KO groups. It was evident that the anti‐senescence marker gene HMGB1 exhibited reduced expression in the ABCA6‐KO group (Figure 5H,K), whereas the senescence marker gene P16 showed increased expression in the ABCA6‐ko group (Figure 5H,L). Similarly, the cell cycle negative regulator TGFB1 was comparatively significantly overexpressed in the WT group (Figure 5I,N), while the cell cycle promoting gene CCND1 showed heightened expression in the ABCA‐KO group (Figure 5I,O). Additionally, cell proliferation was significantly inhibited in the ABCA6‐KO group indicated by much decreased EDU staining (Figure 5J,P). Therefore, we posit that the ABCA6 deficiency accelerates cellular senescence, leading to perturbations in the cell cycle pathway, thereby influencing chondrogenesis and subsequently impacting the initiation and progression of OA.

### 2.6 The Metabolomic Analysis Indicates That the ABCA6 Deficiency Influences Cartilage Formation by Regulating Lipid Metabolism Processes in Chondrocytes

**Figure 6:**
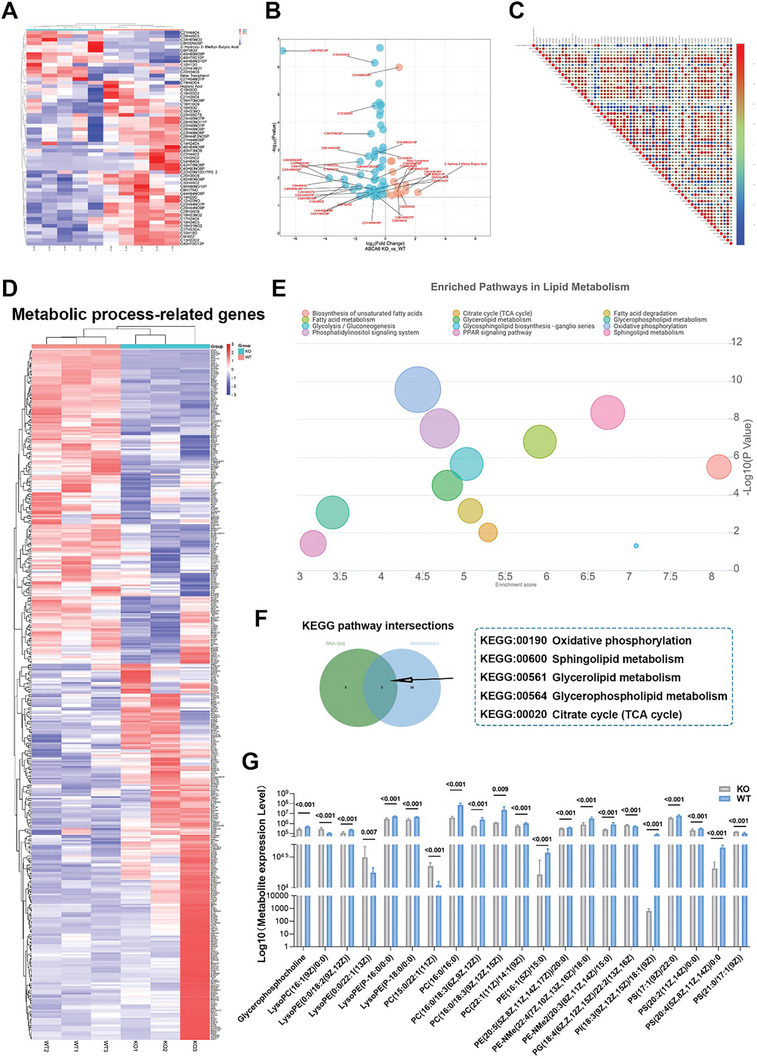
The metabolomic analysis indicates that the ABCA6 gene influences cartilage formation by regulating lipid metabolism processes. A) Volcano Plot of differential metabolite between WT and ABCA6 KO groups. B) Differential metabolite clustering heatmap between WT and ABCA6 KO groups. C) Correlation analysis of differential metabolites between WT and ABCA6 KO groups. D) Heatmap of the expression profiles for metabolic process‐related genes comparing the WT group to the ABCA6 KO group. E) Bubble Chart of Enriched Pathways in Lipid Metabolism. F) Venn diagram of KEGG pathway intersections between RNA‐Seq and metabolomics. G) Metabolite expression level of lipid in metabolomic.

ABCA6 is a member of the A‐family of the ATP binding cassette transporter, which is a group of transmembrane proteins mediating the transmembrane transport of various molecules, and ABCA family is closely related to the homeostasis of lipid. To investigate the metabolic changes in the presence of ABCA6‐KO mice, we conducted further metabolomic analysis of articular chondrocytes from both the WT and ABCA6 KO groups. This analysis revealed significant differences in the metabolites of the ABCA6‐KO group (**Figure** **6**A–C). Furthermore, differential analysis of metabolic process‐related genes in the WT and ABCA6 KO groups (Figure 6D) led us to conclude that impact of ABCA6 gene on cartilage formation is also mediated through the regulation of metabolic processes. Hence, we hypothesize that the ABCA6‐KO group may exhibit significant differences in lipid metabolism, which was confirmed by KEGG analysis of the WT and ABCA6‐KO groups, showing distinct pathways enriched in lipid metabolism in the ABCA6 KO group (Figure 6E). Moreover, there are multiple intersections between the KEGG pathways from RNA‐Seq and Metabolomics (Figure 6F), including oxidative phosphorylation, sphingolipid metabolism, glycerolipid metabolism, glycerophospholipid metabolism, and the citrate cycle (TCA). Additionally, a majority of lipid metabolites showed differences between the WT and ABCA6 KO groups (Figure 6G). In summary, we boldly propose that the ABCA6 deficiency influences cartilage formation by regulating lipid metabolism processes in chondrocytes.

### 2.7 ABCA6 Prevents Joint Degeneration in ABCA6‐KO Mice by Orchestrating Chondrocyte Cholesterol Efflux and Chondrogenesis

**Figure 7:**
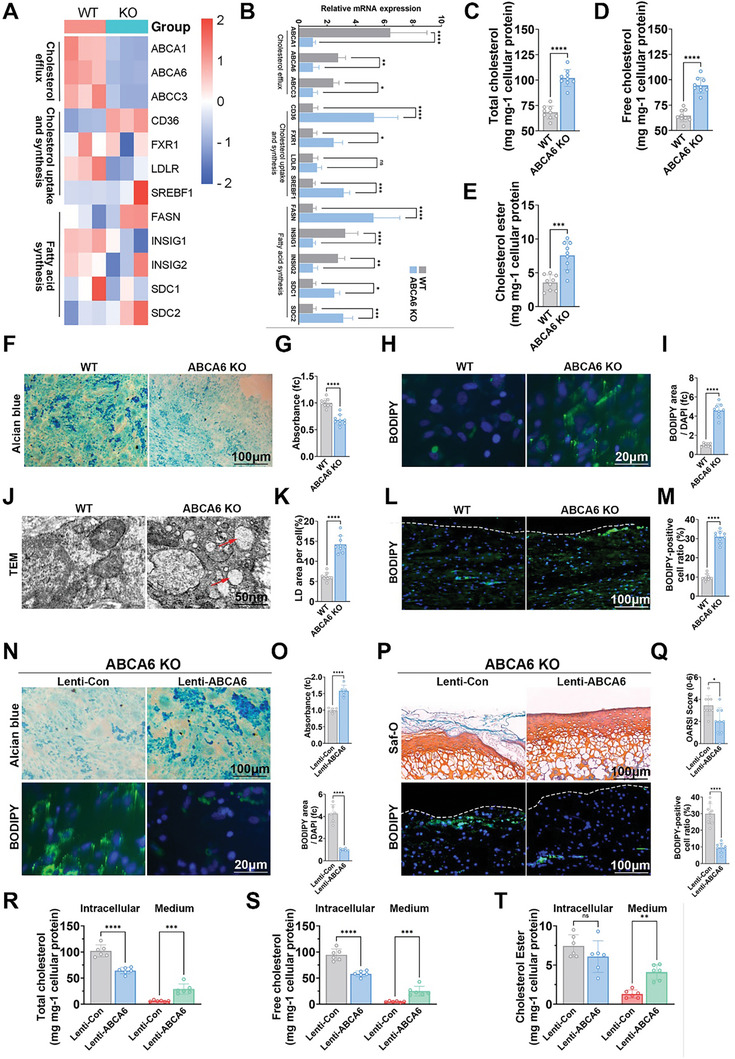
ABCA6 prevents joint degeneration in ABCA6‐KO mice by orchestrating chondrocyte cholesterol efflux and chondrogenesis. A) Heatmap of cholesterol efflux markers, cholesterol uptake markers and synthesis and fatty acid synthesis markers expression profiles within microarray expression profiles in ABCA6 KO group compared to WT group. B) mRNAs expression with ABCA6 KO group compared to WT group was validated with qRT‐PCR for cholesterol efflux‐related mRNAs, cholesterol uptake‐related mRNAs and synthesis and fatty acid synthesis‐related mRNAs. *p < 0.05, **p < 0.01, ***p < 0.001, ****p < 0.0001, NS not significant. C–E) Quantification of total cholesterol (C), free cholesterol (D), and cholesterol ester (E) in ABCA6 KO group compared to WT group. F) Alcian blue staining of mice knee cartilage cells in WT group and ABCA6 KO group. G) Quantification of absorbance in F. H) Bodipy staining of mice knee cartilage cells in WT group and ABCA6 KO group. I) Quantification of bodipy area compared with DAPI in H. J) TEM of mice knee cartilage tissue in WT group and ABCA6 KO group, the red arrow indicates a lipid droplet. K) Quantification of LD area per cell in J. L) Bodipy staining of mice knee cartilage tissue in WT group and ABCA6 KO group. M) Quantification of bodipy‐positive cell ratio in L. N) Alcian blue staining and bodipy staining of mice knee cartilage cells in Lenti‐Con group and Lenti‐ABCA6 group. O) Quantification of absorbance and bodipy area compared with DAPI in N. P) Saf‐O staining and bodipy staining of mice knee cartilage tissue in Lenti‐Con group and Lenti‐ABCA6 group. Q) OARSI score of Lenti‐Con group and Lenti‐ABCA6 group and quantification of bodipy‐positive cell ratio in Q. R–T) Quantification of total cholesterol (R), free cholesterol (S), and cholesterol ester (T) of Lenti‐ABCA6 group compared to Lenti‐Con group with intracellular and medium.

To validate our hypothesis, we focused on KEGG pathways and embarked on a comprehensive analysis within the realm of lipid metabolism including the efflux, uptake and synthesis of cholesterol as well as the fatty acid synthesis pathway. We just posed the differential gene expressions between the WT groups and the ABCA6 KO groups (**Figure** **7**A). Our findings illuminated a diminished expression of marker genes within the cholesterol efflux pathways in the ABCA6‐KO group. This observation was further substantiated through RT‐PCR experiments (Figure 7B), leading us to postulate that ABCA6 plays a pivotal role in the regulation of intracellular cholesterol efflux. Subsequent evaluations of total cholesterol, free cholesterol, and cholesterol esters in both the WT and ABCA6 – KO groups (Figure 7C–E) demonstrated significantly elevated levels of total cholesterol, free cholesterol, and cholesterol esters in the ABCA6 – KO groups. In other words, when cholesterol efflux is restricted, cholesterol accumulates within cells. At this point, both total cholesterol and free cholesterol will increase to varying extents, and the increase in free cholesterol will promote the synthesis of cholesterol esters. The varying degrees of increase in these three substances reflect the situation in cholesterol efflux is blocked. This suggests that ABCA6 deficiency impairs cholesterol efflux, resulting in significantly greater intracellular cholesterol accumulation. Further investigations with Alcian Blue staining of chondrocytes indicated that ABCA6 deficiency was correlated with impaired chondrogenesis with less ECM deposition (Figure 7F,G). Bodipy staining (Figure 7H,I), along with Transmission Electron Microscopy (TEM) observations of chondrocyte in vitro further revealed severe intracellular cholesterol accumulation (Figure 7J,K) in the ABCA6‐KO group. Bodipy staining (Figure 7L,M) of the joint cartilage consistently demonstrated elevated cholesterol levels in vivo in the ABCA6‐KO group. These findings indicated that ABCA6 deficiency led to cholesterol accumulation in chondrocytes. ABCA6 might play a pivotal role in cartilage formation and OA development with orchestrated chondrocyte cholesterol efflux and chondrogenesis. In a subsequent experiment, the reintroduction of the ABCA6 gene (Lenti‐ABCA6 group) into ABCA6‐KO chondrocytes via lentiviral transduction was explored (Figure 7N–Q). With ABCA6 overexpression, the Lenti‐ABCA6 group demonstrated significantly improved chondrogenesis and greater cholesterol efflux in the chondrocytes in vitro (Figure 7N,O). Meanwhile, intra‐articular delivery of ABCA6 lentivirus in the Lenti‐ABCA6 group significantly ameliorated OA development and intracellular cholesterol accumulation in the ABCA6‐KO mice with safranin‐O staining and bodipy staining of the mice joint cartilage tissues (Figures 7P,Q and S3, Supporting Information). Besides, total, free, and esterified cholesterol levels were measured to evaluate the intervention (Figure 7R–T). Subsequent assessments of total cholesterol, free cholesterol, and cholesterol esters within both the Lenti‐Con and Lenti‐ABCA6 groups (Figure 7R–T) revealed significantly lower intracellular cholesterol levels and greater medium cholesterol levels in the Lenti‐ABCA6 groups, suggesting that the ABCA6 overexpression restored cholesterol efflux and ameliorated intracellular cholesterol accumulation in ABCA6‐KO chondrocytes. The results unequivocally demonstrated the reintroduction of ABCA6 with lenti‐ABCA6 could restore cholesterol efflux and, concomitantly recuperated chondrogenesis and prevented OA development in ABCA6‐KO mice.

### 2.8 ABCA6 Overexpression Recuperates Chondrogenesis and Inhibited OA Progression by Restoring Intracellular Cholesterol Balance and Reversing Cellular Senescence in ABCA6‐KO Mice

**Figure 8:**
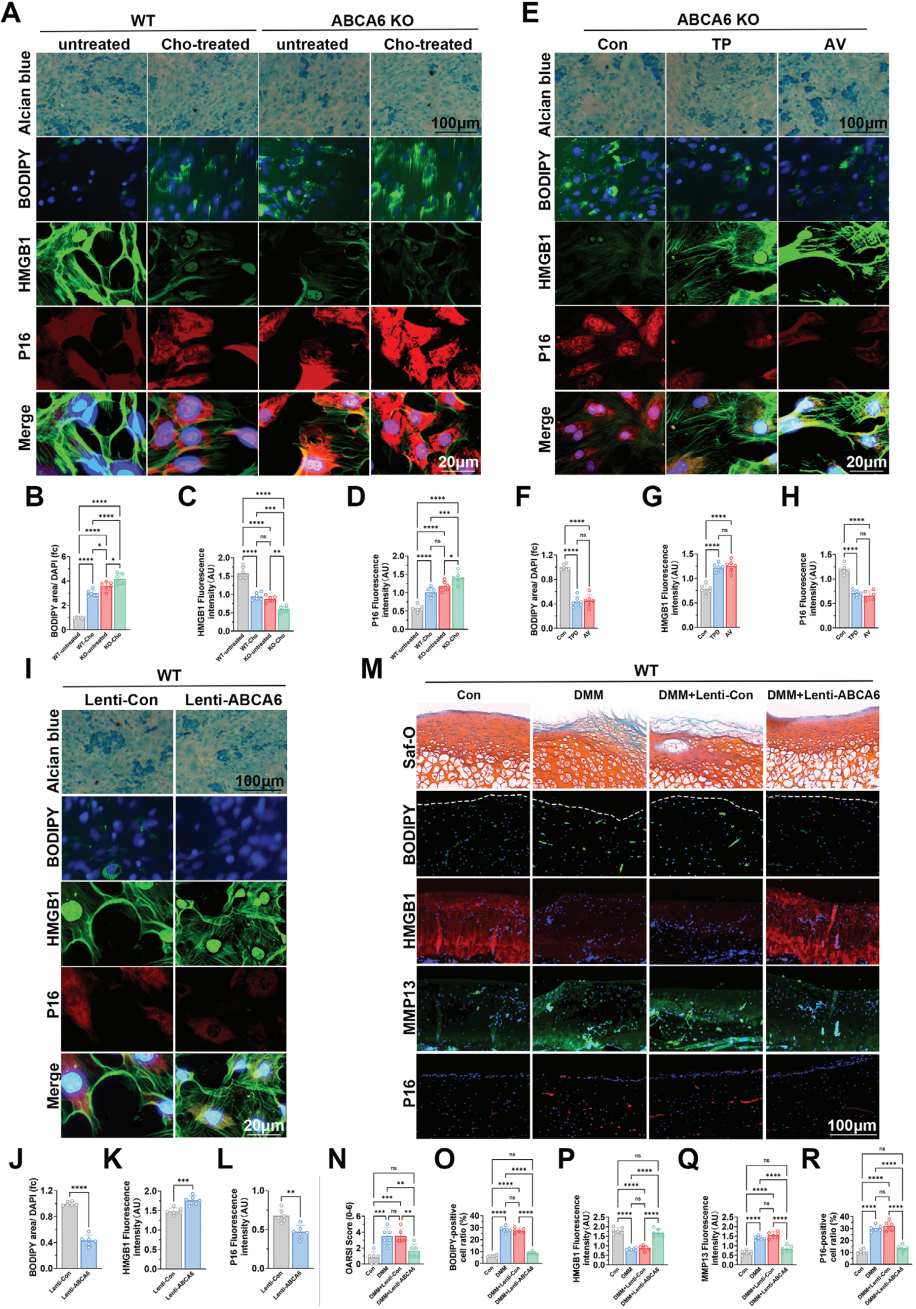
ABCA6 overexpression recuperates chondrogenesis and inhibited OA progression by restoring intracellular cholesterol balance and reversing cellular senescence in ABCA6‐KO mice. A) Alcian blue staining (1st row), bodipy staining (2nd row) and immunofluorescence of HMGB1(3rd row), P16(4th row) and merge (5th row) of mice knee cartilage cells in WT groups and ABCA6 KO groups with cholesterol‐treated (100 µM, 36 h) or untreated. B–D) Quantification of bodipy area compared with DAPI (B), fluorescence intensity of HMGB1 (C) and P16 (D) in A. N = 6 in each group, *p < 0.05, **p < 0.01, ***p < 0.001, ****P < 0.0001, NS not significant. E) Alcian blue staining (1st row), bodipy staining (2nd row) and immunofluorescence of HMGB1(3rd row), P16(4th row) and merge (5th row) of mice knee cartilage cells in ABCA6 KO groups with triparanol (5 µM, 36 h) and atorvastatin (10 µM, 36 h). F–H) Quantification of bodipy area compared with DAPI(F), fluorescence intensity of HMGB1(G) and P16(H) in E. I) Alcian blue staining (1st row), bodipy staining (2nd row) and immunofluorescence of HMGB1(3rd row), P16(4th row) and merge (5th row) of mice knee cartilage cells in Lenti‐Con group and Lenti‐ABCA6 group. J–L) Quantification of bodipy area compared with DAPI(J), fluorescence intensity of HMGB1 (K) and P16 (L) in I. N = 6 in each group, *p < 0.05, **p < 0.01, ***p < 0.001, ****p < 0.0001, NS not significant. M) Saf‐O staining (1st row), bodipy staining (2nd row) and immunofluorescence of HMGB1(3rd row), MMP13(4th row) and P16(5th row) of mice knee cartilage tissue in control group, DMM group, DMM+Lenti‐Con group and DMM+Lenti‐ABCA6. N–R) Quantification of OARSI score (N), bodipy‐positive cell ratio (O), fluorescence intensity of HMGB1 (P), MMP13 (Q), and P16 (R) in M. N = 6 in each group, *p < 0.05, **p < 0.01, ***p < 0.001, ****p < 0.0001, NS not significant.

To elucidate the relationship between cholesterol accumulation and chondrocyte cellular senescence in ABCA6‐KO mice, we conducted a study involving cholesterol treatment of chondrocytes derived from both WT and ABCA6 KO mice groups with untreated groups serving as controls. Alcian Blue staining, Bodipy staining, and immunofluorescence analysis of HMGB1 and P16 expressions were conducted (**Figure** **8**A–D). Our observations revealed a progressive decline in chondrogenic matrix formation across the WT untreated, WT cholesterol‐treated, ABCA6‐KO untreated, and ABCA6 KO cholesterol‐treated groups. This decline was mirrored by a corresponding decrease in HMGB1 expression(Figure 8C) and a significant increase in lipid droplets and P16 expression (Figure 8B,D), These results demonstrated that cholesterol accumulation in the cho‐treated groups could lead to chondrocyte senescence and dampened chondrogenesis in vitro, and ABCA6‐KO further weakened cholesterol efflux and worsened chondrogenesis with accelerated cellular senescence in ABCA6‐KO chondrocytes. Conversely, following the administration of previously described^[^ ^19^ ^]^ lipid‐lowering drugs to the ABCA6 KO groups, namely triparanol (TP) and atorvastatin (AV), an increase in cartilage matrix formation was observed, alongside a significant elevation in HMGB1 expression (Figure 8E,G) as well as a reduction in lipid droplet formation and P16 expression (Figure 8E,F,H) compared to untreated controls. The results unequivocally indicated the lipid‐lowering drugs could recuperate chondrogenesis by restoring intracellular cholesterol balance and reversing cellular senescence in ABCA6‐KO chondrocytes.

Additionally, the overexpression of the ABCA6 gene in the lenti‐ABCA6 chondrocytes via lentiviral transduction resulted in significantly enhanced chondrogenic ECM formation and increased HMGB1 expression (Figure 8I–K). Meanwhile, formation of lipid droplets was significantly inhibited with decreased expression of the senescence marker P16 (Figures 8I–L and S4, Supporting Information) in the Lenti‐ABCA6 group compared to the Lenti‐Control group. To further investigate the therapeutic effects of ABCA6 overexpression in OA progression, intra‐articular delivery of ABCA6 lentivirus was conducted in mice with an OA model. Lenti‐ABCA6 group significantly ameliorated OA development and intracellular cholesterol accumulation in a mice model of OA induced by destabilization of the medial meniscus (DMM) (Figure 8M). Notably, formation of lipid droplets was significantly inhibited with decreased expression of MMP13 and P16 gene in the DMM+Lenti‐ABCA6 group (Figure 8M–R), alongside a significant increase in HMGB1 expression compared to the DMM+Lenti‐Con group (Figure 8P). These experiments conclusively demonstrate that the ABCA6 overexpression could serve as a potential target in OA treatment by promoting intracellular cholesterol efflux and delaying chondrocyte cellular senescence in mice.

## 3 Discussion

In this study, we conducted WES across a four‐generation lineage presenting with six cases of patellar dysplasia, complemented by genetic linkage analysis. We identified six mutational sites within the genes B3GNT1, DRAP1, ABCA10, ABCA6, and SYNJ1. Subsequently, Sanger sequencing and protein function prediction analyses pinpointed a potential pathogenic variant, ABCA6 D797N, the gene mutation that was implicated in the condition of familial patellar dysplasia. To assess the ubiquity of this mutation's impact and the role of the ABCA6 gene in cartilage, we conducted histomorphological analyses on various types of patellar dislocation. A consistent downregulation of ABCA6 expression was observed; moreover, it was correlated with chondrocyte degeneration and subsequent OA. To establish the existence of a linear relationship rather than a coincidental one, further investigations involved the development of an ABCA6‐KO mice model, through a suite of in vivo experiments, this model demonstrated severe knee joint degeneration and significant accelerated OA progression, thus corroborated the preliminary findings. Besides, with breeding ABCA6 KO mice with WT mice counterparts, we extracted SMSCs from their progeny mice. These SMSCs were then cultivated in a 3D environment to create a chondro‐organoid model. Comparative analyses between normal and aberrant chondro‐organoids confirmed that ABCA6 deficiency impairs chondrogenesis and reduces the deposition of extracellular matrix within cartilage nodules, generating premature chondrocyte senescence and degeneration. Delving deeper into the mechanistic role of ABCA6 in cartilage, we performed mRNA sequencing and metabolomic profiling, the findings of which led us to hypothesize that ABCA6 deficiency orchestrates cellular senescence and lipid metabolism in chondrocytes. Together with subsequent in vitro and in vivo experiments, we demonstrated that the ABCA6 gene plays a pivotal role in cartilage formation by promoting cholesterol efflux in metabolic pathways, thereby modulating cellular senescence and ultimately enhancing the cartilage matrix deposition and fostering chondrogenesis.

Multiple prior studies have demonstrated a strong association between patellar dysplasia and patellar dislocation, as well as the development of OA.^[^ ^1^, ^2^, ^3^, ^4^ ^]^ Although we did not test the expression of ABCA6 in clinical specimens from OA patients, the diminished expression of ABCA6 in patellar dysplasia and patellar dislocation undoubtedly suggests its relevance in joint‐related diseases. In our investigation into the correlation between ABCA6 and cartilage development, we utilized 3D‐cultured SMSCS to engineer cartilage organoids in vitro. Cartilage, one of the most challenging tissues to regenerate,^[^ ^20^ ^]^ has been developed into the cell‐engineered cartilage organoids. Its usage to better simulate the chondral environment has gained traction for the development of personalized therapeutic strategies aimed at cartilage‐related diseases.^[^ ^21^ ^]^ Commonly employed cell types for the construction of cartilage organoids include embryonic stem cells, induced pluripotent stem cells, mesenchymal stem cells, and human periosteum‐derived cells.^[^ ^22^ ^]^ In our previous research, we have successfully employed 3D‐cultured SMSCS to construct cartilage organoids, which have exhibited superior chondral characteristics compared to organoids constructed by other cell types such as bone marrow‐derived mesenchymal stem cells (BMSC) and human induced pluripotent stem cells (hiPSCs).^[^ ^23^ ^]^ This approach enables us to closely replicate in vitro the chondral environment in vivo, thus enhancing the reliability of our experimental outcomes. This corroborates the reliability of this experimental method of cartilage formation in vitro, thereby solidifying and innovating subsequent research and applications of organoids in chondrogenesis and OA.

OA is one of the most prevalent joint diseases and often manifests in the advanced stages of various joint disorders.^[^ ^24^, ^25^, ^26^ ^]^ According to the studies published so far, cellular senescence remains the most common contributing factor yielding the occurrence of OA.^[^ ^27^, ^28^, ^29^ ^]^ Concurrently, there also exists a close relationship between cellular senescence and metabolism.^[^ ^14^, ^30^, ^31^ ^]^ As a result, research on the interplay among metabolism, senescence, and OA has garnered considerable attention. However, many studies have focused on mitochondrial metabolism in regulating senescence and OA.^[^ ^32^, ^33^, ^34^ ^]^ Regarding cholesterol, previous research has demonstrated that all mammalian cell membranes contain cholesterol to maintain membrane integrity. Senescence involves changes in cholesterol metabolism of cell. Some of these changes may either promote or inhibit the progression of age‐related diseases during senescence, particularly Alzheimer's disease.^[^ ^35^ ^]^ While the association between cholesterol metabolism and OA has been established,^[^ ^36^ ^]^ the role of cholesterol metabolism in the mechanism of OA pathogenesis remains inadequately explored.^[^ ^37^, ^38^, ^39^ ^]^ Previous researches on cholesterol metabolism and OA have been largely confined to studies targeted at lipid‐lowering drugs to alleviate osteoarthritis, which yield less than satisfactory results.^[^ ^36^, ^40^, ^41^, ^42^ ^]^ Apart from this, recent studies have centered on the cholesterol–CH25H–CYP7B1–RORα axis^[^ ^19^ ^]^ as well as the cholesterol‐LRP3‐syndecan‐4 axis.^[^ ^43^ ^]^ Regrettably, the upstream components of these axes are still cholesterol, and regulating cholesterol metabolism to counteract OA has not yet been proposed as new therapeutic targets. However, our WES analysis of clinical samples and subsequent research has led us to identify novel gene targets. Although the relationship between ABCA6 and cholesterol has been explored,^[^ ^44^, ^45^ ^]^ the investigation into the association between ABCA6 and OA represents a novel direction. We have not only elucidated the role of ABCA6 in regulating cholesterol efflux to promote cartilage development, but also, through rigorous experimental approaches, established that ABCA6 orchestrates cholesterol efflux in a manner that impacts cellular senescence, ultimately fostering cartilage development. This thorough exploration of the lipid metabolism mechanism of ABCA6 expands the scope of research on chondrocyte metabolism and its relationship with cellular senescence.

In summary, a disease‐causing mutation of ABCA6 was identified for FPD. ABCA6 was correlated with PD occurrence and subsequent OA progression. ABCA6 could serve as a potential target in chondrogenesis and OA treatment by orchestrated intracellular cholesterol efflux and delayed cellular senescence. Ultimately, our research provides new potential targets for PD and OA treatment, paving the way for future therapeutic strategies and further clinical translations.

## 4 Experimental Section

### Patients

TPD (23.3 ± 10.8 years, three males and 83 females), SPD (32.4 ± 6.3 years, three males and three females), and FPD patients were consecutively recruited from the department of orthopedics of The First Affiliated Hospital with Nanjing Medical University. Controls (42.2 ± 4.5 years, three males and three females) were recruited from amputee patients. The knee cartilage samples were obtained from the patella. TPD, SPD, and FPD were diagnosed according to clinical criteria and radiographic evidence. All controls were confirmed to have no symptom or history of patellar dislocations. Subjects with any genetic disease except FPD were excluded. All the subjects were Han Chinese living in or around Nanjing. The ethical committee for The First Affiliated Hospital of Nanjing Medical University approved all procedures, and patients’ informed consent forms were obtained. All methods were performed in accordance with the relevant guidelines and regulations (No. 2022‐SR‐504).

### Whole Exome Sequencing, Linkage Analysis, Sanger Sequencing and Protein Function Simulation

Agilent SureSelect Human All Exon 50Mb on an Illumina Hiseq2500 instrument was used to perform WES for two patients (F003, F009) diagnosed with patellar dysplasia confirmed by imaging and for one normal control (F005) in the study family, and a series of filters were applied. Meanwhile, a genetic linkage analysis of the whole genome was conducted for all the 12 family members using Illumina Omni ZhongHua‐8 V2 BeadChip. The chromosomal region with LOD >1 was identified using log‐odds score. Combining the results of WES and linkage analysis results, the possible exon mutation sites in the chromosomal region with LOD >1 were identified. These mutations were then verified through in situ Sanger sequencing in the family members. After that, the verified mutations were rechecked in another 200 independent Chinese normal controls to exclude gene polymorphism. Finally, for the remaining candidate mutation sites, the potential influence of the mutations on protein functions was predicted using Poly‐Phen© and Mutation‐Taster© to help determine the most possibly responsible mutation site(s) for patellar dysplasia in the study family.

### Immunofluorescence Staining

Initially, tissues were fixed in 4% PFA, permeabilized with PBS containing 0.5% Triton X‐100 for 20 min, and subsequently blocked with a solution of 3% BSA containing 0.025% Triton X‐100 and 5% FBS at room temperature for 30 min. The tissues were then subjected to immunostaining by incubating them with primary antibodies at 4 °C overnight. Subsequently, the tissues were washed three times with PBS and then incubated with suitable secondary antibodies. Following another round of washing, DAPI was used for nuclear counterstaining for 5 min. The visualization of immunofluorescence was performed using a confocal microscope (Carl Zeiss, Germany). Each experiment was performed in triplicate, and representative images from confocal microscopy are presented.

### Animals

ABCA6 KO mice and Wildtype C57Bl/J6 were acquired from GemPharmatech Co., Ltd (Nanjing, China). The First Affiliated Hospital of Nanjing Medical University's Animal Committee gave its approval to all of the experiments (No. IACUC‐2303016).

### Construction of Mice Model of OA

After the skin incision, a medial parapatellar incision was applied and the patella was dislocated. Anterior cruciate ligament transection was performed to construct an OA model. After the operation, mice were allowed to move freely in their single cages and fed with standard food and water. Serial sections (4‐µm thick) were cut sagittally through the center of the most diseased osteoarthritic site and stained with H&E and Saf‐O stainings according to standard protocols. Histological assessment of sagittal sections of the knee joints was conducted by two blinded observers who followed the Osteoarthritis Research Society International (OARSI) scoring system. Measurements were also performed for osteophyte maturation, synovitis score (0–3, 0 = no synovial thickening; 1 = lining of two cell layers; 2 = several extra cell layers; 3 = clear inflammation with cell infiltrate or exudate), and subchondral bone plate thickness (the region between the osteochondral junction and marrow space on the medial side of the tibial plateau). After the operation, mice were allowed to move freely in their single cages and fed with standard food and water.

### DMM Mice

Anesthetized the mice, performed a medial incision over the knee joint to expose the joint capsule, and then transected the anterior horn of the medial meniscus. Sham‐operated mice were used as controls.

### Cell Isolation

Chondrocytes: The knee joint cartilage tissue (enclosed in the knee joint capsule) was obtained from mice. It was thoroughly disinfected with iodine tincture and immersed in sterile physiological saline. Then, the tissue is transferred to a sterile laminar flow hood for strict disinfection, and the surrounding tissues that enclose the cartilage were removed. Subsequently, it was washed three times with sterile PBS (Phosphate Buffered Saline). The cartilage tissue was cut into pieces and placed in a 6 cm cell culture dish. 0.25% EDTA‐Trypsin was used for digestion for 2 h. Subsequently, DMEM (Dulbecco's Modified Eagle Medium) medium containing 10% Fetal Bovine Serum (FBS) was added to terminate the digestion process. Then, the mixture was pipetted and transferred to a centrifuge tube, which was centrifuged at 400 g for 5 min to remove the supernatant. Next, 0.2% Type II collagenase was added, and the mixture was digested overnight in an incubator at 37 °C and 5% CO₂. The digestion was terminated by adding DMEM medium containing 10% FBS with the same volume as the collagenase. Then, the cell suspension was pipetted and filtered through a 70 µm cell filter into a centrifuge tube, which was centrifuged at 400 g for 5 min. The resulting cell pellet was inoculated into a new cell culture dish and incubated in an incubator at 37 °C and 5% CO₂ for subsequent experiments.

SMSC: The synovial tissue was taken out from the knee joint cavity and soaked in double – antibiotic H – DMEM containing 1000 U mL⁻^1^ streptomycin and penicillin. It was transferred at low temperature (using an ice box) and then moved into a super – clean workbench. The synovial tissue was rinsed three times with sterile PBS containing 1000 U mL⁻^1^ double – antibiotic. It was cut into tissue pieces of ≈1 mm × 1 mm in size with ophthalmic scissors. Tenfold volume of 0.4% type I collagenase was added, and digestion was carried out for 4 h under the conditions of 37 °C, 5% CO₂ by volume and saturated humidity. After most of the floccules disappeared, the mixture was filtered through a 100 – mesh copper net. The filtrate was aspirated and centrifuged at 1200 rpm for 5 min. The supernatant was discarded, and the tissue was washed three times with sterile PBS. Using high – glucose H – DMEM medium containing 10% fetal bovine serum by volume (containing 200 mmol L⁻^1^ glutamine, 100 µmol L⁻^1^ ascorbic acid, 100 U mL⁻^1^ penicillin, and 100 mg L⁻^1^ streptomycin), cells were inoculated into the culture dish at a cell concentration of 2 × 10⁵ cm^2^ and cultured in an incubator at 37 °C and 5% CO₂ by volume. The medium was changed for the first time 48 h later, and then the medium was changed every 48 h. When the cells reached 80% – 90% confluence, the culture medium in the culture dish was removed, and the cells were washed twice with PBS to wash away cell metabolites and apoptotic cells. After gently shaking with 2.5 g L⁻^1^ trypsin containing EDTA (2 mL is added into a 10 – cm dish), the cells were digested at 37 °C for 1 min. Then, they were observed under a microscope. When the cytoplasm shrinked and the intercellular substance increased, the culture medium containing 10% fetal bovine serum by volume was added to terminate the digestion. The adherent cells were gently and repeatedly pipetted until they were suspended. After centrifugation at 1000 rpm for 5 min, the supernatant was discarded, and the cells were washed twice with PBS. Subculture was carried out at a ratio of 1:3 for subsequent experiments.

### Cell Expansion

SMSCs and chondrocyte were isolated from different types of mice. These cells were cultured and proliferated in Dulbecco's Modified Eagle Medium (DMEM supplied by Life Technologies in China), which was enriched with 10% fetal bovine serum (FBS sourced from HyClone, a part of Thermo Scientific in the USA), 1% combined antibiotic‐antimycotic solution (containing 100 units/mL penicillin, 100 µg mL⁻^1^ streptomycin, and 0.25 µg mL⁻^1^ amphotericin B), and 1 mM sodium pyruvate (also from Life Technologies, China). SMSCs and chondrocyte were maintained at optimal conditions of 37 °C, 5% carbon dioxide, and 95% relative humidity until they reached the third passage. Nutrient media were refreshed at intervals of two to three days. When cell growth reached 80–90% confluence, cells were detached using trypsin (provided by Life Technologies, China). This enzymatic treatment was consistently employed for both subculturing and collecting cells throughout the experimental process.

### Formation of SMSC Organoids

Agarose at a 3% (w/v) concentration (sourced from Invitrogen, China) was cast onto a master mold fabricated from polydimethylsiloxane (PDMS, specifically the Corning Sylgard 184 elastomer from MAVOM Chemical Solutions), which featured pillars of 200 µm in diameter. Once the agarose solidified, microwell inserts, each covering ≈1.8 cm2, were excised and arranged into 24‐well plates. Subsequently, each well was filled with 1 mL of phosphate‐buffered saline (PBS), followed by a 30‐min UV sterilization process. These inserts were designed to house ≈2000 microwells. SMSCs were collected and deposited into the wells at a density of 1 000 000 cells per well, aiming to achieve a rough estimate of 500 cells within each organoid post self‐aggregation. The SMSC organoids were then nurtured to form cartilage‐like microtissues within a serum‐free chondrogenic medium (CM) that was chemically defined. This medium consisted of low glucose DMEM (LG‐DMEM from Gibco) supplemented with 1% antibiotic‐antimycotic solution (100 units/mL penicillin, 100 µg mL⁻^1^ streptomycin, and 0.25 µg mL⁻^1^ amphotericin B), 1 mM ascorbate‐2 phosphate, 100 nM dexamethasone, 40 µg mL⁻^1^ proline, and ITS+ Premix Culture Supplement (from Corning), which includes insulin, transferrin, selenious acid, bovine serum albumin (BSA), and linoleic acid at specified concentrations. Additionally, the medium contained 100 ng mL⁻^1^ growth/differentiation factor 5 (GDF5 from PeproTech) and 10 ng mL⁻^1^ transforming growth factor‐beta 3 (TGF‐β3 from PeproTech). The medium was half‐refreshed every two to three days.

### RNA Isolation, cDNA Synthesis, and qRT‐PCR

Total RNA was extracted from joint tissues or BMSCs utilizing TRIzol reagent (Invitrogen, Carlsbad, CA, USA) as per the protocol provided by the manufacturer. The evaluation of RNA integrity and concentration was conducted using both a bioanalyzer (Agilent Inc., Santa Clara, CA, USA) and a nanodrop spectrophotometer (Thermo Scientific). For the quantitative analysis of miRNA and mRNA, RT‐PCR was executed using a qSYBR‐green‐containing PCR kit (Qiagen, Germantown, MD, USA) in conjunction with an RT‐PCR system (Applied Biosystems, SA, USA). The normalization of qRT‐PCR data was achieved using housekeeping genes, namely U6 small nuclear RNA (snRNA) and GAPDH. The specific qRT‐PCR primers for both mRNA and miRNA, along with the internal control, were sourced from Applied Biosystems. The PCR assays were conducted in a triplicate set‐up and analyzed employing the 2−△△Ct method. Requests for the sequences of all primers can be directed to the authors.

### Microarray Analysis

We conducted microarray analysis on six cartilage samples (mRNA microarray for 3 WT and 3ABCA6 KO) using the Agilent mRNA Microarray Kit, Release 21.0, 8×60K (Agilent Technologies, CA, USA). Total RNA was quantified using a NanoDrop ND‐2000 (Thermo Scientific, SA, USA), and RNA integrity was assessed with an Agilent Bioanalyzer 2100 (Agilent Technologies). Following the manufacturer's protocols, sample labeling, microarray hybridization, and washing were performed. Total RNA was dephosphorylated, denatured, and then labeled with cyanine‐3‐CTP. After purification, the labeled RNAs were hybridized to the microarray. Subsequently, the arrays were scanned using an Agilent Scanner G2505C (Agilent Technologies). Feature Extraction software (version 10.7.1.1, Agilent Technologies) was used to analyze the array images and obtain raw data. Genespring software (version 14.8, Agilent Technologies) was utilized for the basic analysis of the raw data. The raw data were normalized using the quantile algorithm. Probes with at least 100.0 percent of samples in any one condition out of two conditions with flags in “Detected” were chosen for further data analysis. Differentially expressed miRNAs and mRNAs were identified using R software (Version 3.6.1) with the “limma” package through fold change (FC) and adjusted P value. The threshold for up‐ and down‐regulated genes was set at an FC >4.0 and an adjusted P value <0.05. The differentially expressed mRNAs were validated by quantitative reverse transcriptase–polymerase chain reaction (qRT‐PCR). Target genes of the candidate miRNA were identified based on the intersection between differentially expressed mRNAs and different predicted algorithms (Funrich software and five online predicting databases including miRDB, mRanda, TargetScan, PITA, and PicTar). GO analysis and KEGG analysis were applied to determine the roles of these target genes by R. Hierarchical clustering was performed to show the differential mRNA expression patterns among samples using scatter plots, volcano plots, and heatmaps for visualization.

### Metabolomics Analysis

An ACQUITY UPLC I‐Class plus(Waters Corporation, Milford, USA) fitted with Q‐Exactive mass spectrometer equipped with heated electrospray ionization (ESI) source (Thermo Fisher Scientific, Waltham, MA, USA) was used to analyze the metabolic profiling in both ESI positive and ESI negative ion modes. An ACQUITY UPLC HSS T3 column (1.8 µm, 2.1 × 100 mm) was employed in both positive and negative modes. The binary gradient elution system consisted of (A) water (containing 0.1% formic acid, v/v) and (B) acetonitrile (containing 0.1% formic acid, v/v) and separation was achieved using the following gradient: 0.01 min, 5% B; 2 min, 5% B; 4 min, 30% B; 8 min, 50% B; 10 min, 80% B; 14 min, 100% B; 15 min, 100% B; 15.1 min, 5% and 16 min, 5%B. The flow rate was 0.35 mL min^−1^ and column temperature was 45 °C. All the samples were kept at 10 °C during the analysis. The injection volume was 1 µL. The mass range was from m/z 100 to 1000. The resolution was set at 70 000 for the full MS scans and 17 500 for HCD MS/MS scans. The Collision energy was set at 10, 20, and 40 eV. The mass spectrometer operated as follows: spray voltage, 3800 V (+) and 3200 V (−); sheath gas flow rate, 35 arbitrary units; auxiliary gas flow rate, eight arbitrary units; capillary temperature, 320 °C; Aux gas heater temperature, 350 °C; S‐lens RF level, 50.

The original LC‐MS data were processed by software Progenesis QI V2.3 (Nonlinear, Dynamics, Newcastle, UK) for baseline filtering, peak identification, integral, retention time correction, peak alignment, and normalization. Main parameters of 5 ppm precursor tolerance, 10 ppm product tolerance, and 5% product ion threshold were applied. Compound identification was based on precise mass‐to‐charge ratio (M/z), secondary fragments, and isotopic distribution using The Human Metabolome Database (HMDB), Lipidmaps (V2.3), Metlin, and self‐built databases. The extracted data were then further processed by removing any peaks with a missing value (ion intensity = 0) in more than 50% in groups, by replacing zero value by half of the minimum value, and by screening according to the qualitative results of the compound. Compounds with resulting scores below 36 (out of 60) points were also deemed to be inaccurate and removed. A data matrix was combined from the positive and negative ion data. The matrix was imported in R to carry out Principle Component Analysis (PCA) to observe the overall distribution among the samples and the stability of the whole analysis process. Orthogonal Partial Least‐Squares‐Discriminant Analysis (OPLS‐DA) and Partial Least‐Squares‐Discriminant Analysis (PLS‐DA) were utilized to distinguish the metabolites that differ between groups. To prevent overfitting, sevenfold cross‐validation and 200 Response Permutation Testing (RPT) were used to evaluate the quality of the model. Variable importance of Projection (VIP) values obtained from the OPLS‐DA model were used to rank the overall contribution of each variable to group discrimination. A two‐tailed Student's T‐test was further used to verify whether the metabolites of difference between groups were significant. Differential metabolites were selected with VIP values greater than 1.0 and p‐values less than 0.05. Differential metabolites were further used to for KEGG pathway (http://www.genome.jp/kegg/) enrichment analysis.

### Cholesterol Assay

Cellular total cholesterol levels were measured using a Total Cholesterol Assay Kit (Cell Biolabs). In brief, cellular lipids were extracted using chloroform:2‐propanol:NP‐40 (7:11:0.1) in a micro‐homogenizer, and the levels of total cholesterol and free cholesterol were determined according to the manufacturer's instructions. The amount of cholesterol ester was calculated by subtracting the amount of free cholesterol from the amount of total cholesterol.

### Lentivirus

In a 24‐well culture plate, once chondrocytes had reached 50–60% confluency, 400 µL of medium, 20 µL of lentivirus (Lenti‐ABCA6 or Lenti‐control), and 2.25 µg of Polybrene were added to each well. Incubated at 37 °C with 5% CO2 for 24 h. After this incubation period, the medium was replaced with complete medium as mentioned above and continued culturing for an additional 48 h. Finally, the cells were collected and the expression of ABCA6 was assessed using RT‐PCR and WB.

Intra‐articular injections into the mice knee (once weekly for 3 weeks) of lentivirus (Lenti‐ABCA6, 1 × 10^9^ plaque forming units (PFU) in a total volume of 10 µl), intra‐articular injection of empty adenovirus (Lenti‐control) was used as a control, sham‐ or OA‐operated mice underwent intra‐articular injections of lentivirus 10 days post‐operation. All lentiviruses were purchased from Vector Biolabs.

### Statistical Analysis

Statistical analysis was conducted using SPSS software (Version 19.0, SPSS Inc., Chicago, IL, USA) and GraphPad Prism (Version 8.0, GraphPad Software Inc., San Diego, CA, USA). Data analysis was performed using appropriate statistical tests including Mann–Whitney U‐test, Student's t‐test, and one‐way ANOVA. A significance level of P <0.05 was used for determining statistical significance.

### Ethical Approval

The ethical committee for The First Affiliated Hospital of Nanjing Medical University approved all procedures (No. 2022‐SR‐504).

## Conflict of Interest

The authors declare no conflict of interest.

## Author Contributions

Y.W., Q.W., and Y.‐Q.Y. contributed equally to conceiving the study and designing the experiments. W.‐B.J. helped perform the animal experiments. Y.S. and P.‐L.F. analyzed the data and wrote the manuscript. Y.S. and K.‐R.D. helped edit the manuscript and provided oversight. All authors read and approved the final manuscript.

## Supporting information



Supporting Information

Supporting Information

Supporting Information

## Data Availability

All data associated with this study are present in the paper or the supplementary materials.
