# Supplementary material for: ABCA6 Regulates Chondrogenesis and Inhibits Joint Degeneration via Orchestrated Cholesterol Efflux and Cellular Senescence
Source: Adv Sci (Weinh). 2025 Jan 17;12(10):2410414. doi: 10.1002/advs.202410414 (PMC11904997; doi:10.1002/advs.202410414)
Supplement: Supplementary file 1 — Supporting Information [file ADVS-12-2410414-s002.docx]

**Supplementary Materials**

**
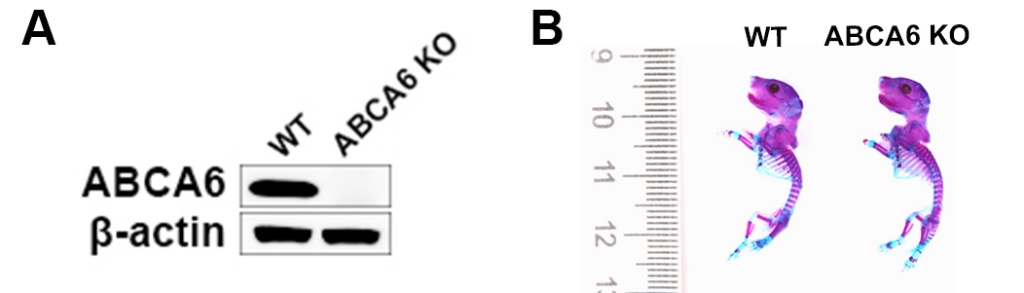
**

**Figure S1. A.** Identification of ABCA6-KO mice with WB staining compared to WT mice. **B.** Saf-O & fast green staining of the whole body skeleton was performed for neonatal ABCA6-KO mice compared to WT mice.


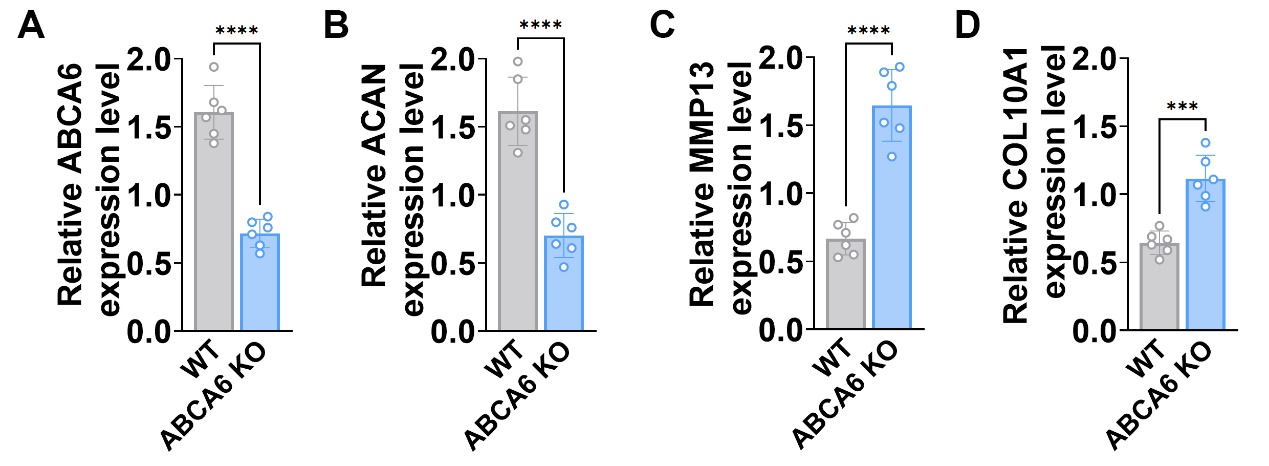
**Figure S2**. Quantitative fluorescence intensity of ABCA6(A), ACAN(B), MMP13(C), COL10A1(D) in Figure 3E. N=6 in each group, ***P < 0.001, ****P < 0.0001.


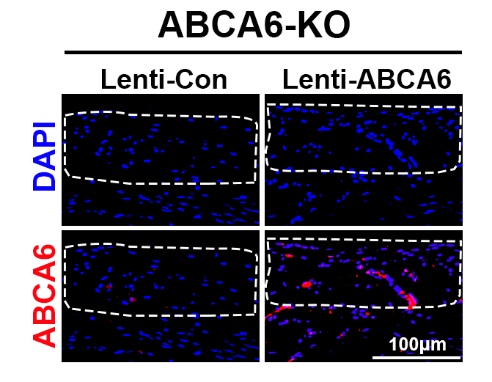


**Figure S3**. Successful overexpression of ABCA6 into cartilage tissue with ABCA6 (red) and DAPI(blue) staining.


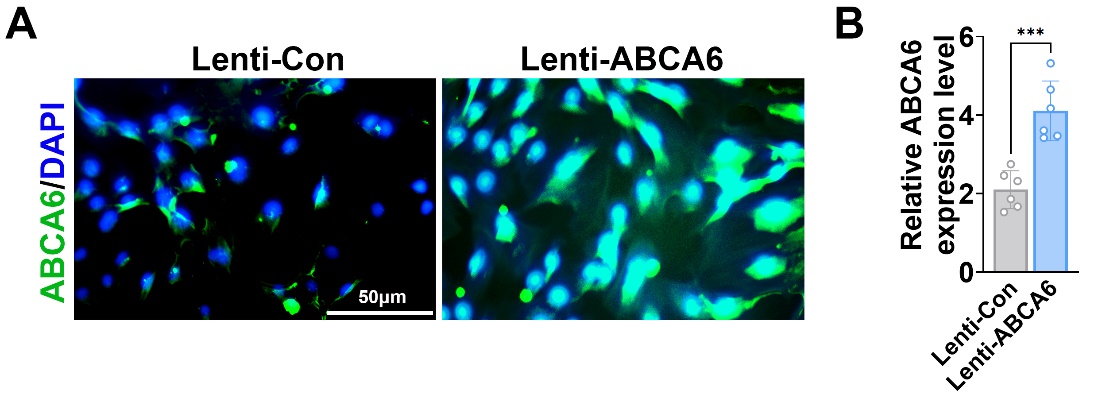


**Figure S4**. **A.** Successful overexpression of ABCA6 in chondrocytes with ABCA6 staining(green) and DAPI(blue). **B.** Successful overexpression of ABCA6 in chondrocytes with qPCR for ABCA6 expression. N=6 in each group, ***P < 0.001
