# Supplementary material for: ABCA6 Regulates Chondrogenesis and Inhibits Joint Degeneration via Orchestrated Cholesterol Efflux and Cellular Senescence
Source: Adv Sci (Weinh). 2025 Jan 17;12(10):2410414. doi: 10.1002/advs.202410414 (PMC11904997; doi:10.1002/advs.202410414)
Supplement: Supplementary file 3 — Supporting Information [file ADVS-12-2410414-s001.docx]

**Table S2** Inheritance of Candidate Variants

| Chromosome | Initiation Site | Pause Site | Original Base | Mutant Base | Hom/Het | Gene Region | Gene | Mutation Type | Protein Impact |
| --- | --- | --- | --- | --- | --- | --- | --- | --- | --- |
| **chr11** | **66114017** | **66114017** | **T** | **G** | **het** | **exonic** | **B3GNT1** | **NS-SNV** | **K334Q** |
| **chr11** | **65687908** | **65687908** | **A** | **G** | **het** | **exonic** | **DRAP1** | **NS-SNV** | **M102V** |
| **chr17** | **67103837** | **67103837** | **C** | **T** | **het** | **exonic** | **ABCA6** | **NS-SNV** | **D797N** |
| chr17 | 67145196 | 67145197 | AG | - | het | exonic | ABCA10 | F-D | 1504del |
| chr17 | 67190540 | 67190543 | GACA | - | het | exonic | ABCA10 | F-D | 443_444del |
| chr21 | 34003929 | 34003929 | - | GATTTA | het | exonic, splicing | SYNJ1 | NF-I | V1405delinesVNT |

Hom, homozygote; Het, heterozygote; NS-SNV, nonsynonymous single nucleotide variant; F-D, frameshift deletion; NF-I, nonframeshift insertion

**Table S3** Protein Conservation Level for Non-Synonymous Changes D797N in ABCA6 Gene

| Species | Match | Gene | AA | Alignment |
| --- | --- | --- | --- | --- |
| Human | all conserved | ABCA6 | 797 | M K L E G Q S T I E Q D F E Q V E |
| Mutated | all conserved | ABCA6 | 797 | M K L E G Q S T I E Q N F E Q V E |
| Ptroglodytes | all identical | ABCA6 | 729 | M K L E G Q S T I E Q D F E Q V E |
| Mmulatta | all identical | ABCA9 | 796 | M K L E G Q S T I E Q D F K Q V E |
| Fcatus | all identical | ABCA6 | 796 | M K L E G K P T I E Q D F E Q A E |
| Mmusculus | all identical | ABCA6 | 797 | L N L E G E P S T K Q D F E K R E |

AA, amino acid.
